# Frustration With Technology and its Relation to Emotional Exhaustion Among Health Care Workers: Cross-sectional Observational Study

**DOI:** 10.2196/26817

**Published:** 2021-07-06

**Authors:** Daniel S Tawfik, Amrita Sinha, Mohsen Bayati, Kathryn C Adair, Tait D Shanafelt, J Bryan Sexton, Jochen Profit

**Affiliations:** 1 Department of Pediatrics Stanford University School of Medicine Palo Alto, CA United States; 2 Operations, Information, and Technology Stanford University Graduate School of Business Stanford, CA United States; 3 Department of Biomedical Informatics Stanford University School of Medicine Stanford, CA United States; 4 Duke Center for Healthcare Safety and Quality Duke University Health System Durham, NC United States; 5 Department of Medicine Stanford University School of Medicine Stanford, CA United States; 6 WellMD Center Stanford University School of Medicine Stanford, CA United States; 7 Department of Psychiatry Duke University School of Medicine Duke University Health System Durham, NC United States; 8 California Perinatal Quality Care Collaborative Palo Alto, CA United States

**Keywords:** frustration with technology, emotional exhaustion, professional burnout, work-life integration, biomedical technology, work-life balance, user-centered design, electronic health records, medical informatics applications, health information systems

## Abstract

**Background:**

New technology adoption is common in health care, but it may elicit frustration if end users are not sufficiently considered in their design or trained in their use. These frustrations may contribute to burnout.

**Objective:**

This study aimed to evaluate and quantify health care workers’ frustration with technology and its relationship with emotional exhaustion, after controlling for measures of work-life integration that may indicate excessive job demands.

**Methods:**

This was a cross-sectional, observational study of health care workers across 31 Michigan hospitals. We used the Safety, Communication, Operational Reliability, and Engagement (SCORE) survey to measure work-life integration and emotional exhaustion among the survey respondents. We used mixed-effects hierarchical linear regression to evaluate the relationship among frustration with technology, other components of work-life integration, and emotional exhaustion, with adjustment for unit and health care worker characteristics.

**Results:**

Of 15,505 respondents, 5065 (32.7%) reported that they experienced frustration with technology on at least 3-5 days per week. Frustration with technology was associated with higher scores for the composite Emotional Exhaustion scale (*r*=0.35, *P*<.001) and each individual item on the Emotional Exhaustion scale (*r*=0.29-0.36, *P*<.001 for all). Each 10-point increase in the frustration with technology score was associated with a 1.2-point increase (95% CI 1.1-1.4) in emotional exhaustion (both measured on 100-point scales), after adjustment for other work-life integration items and unit and health care worker characteristics.

**Conclusions:**

This study found that frustration with technology and several other markers of work-life integration are independently associated with emotional exhaustion among health care workers. Frustration with technology is common but not ubiquitous among health care workers, and it is one of several work-life integration factors associated with emotional exhaustion. Minimizing frustration with health care technology may be an effective approach in reducing burnout among health care workers.

## Introduction

Technological innovation has expanded the horizons of medicine in recent decades [1], but these advances have been accompanied by increased clerical burden among physicians and other health care providers. Electronic health record (EHR) adoption has improved emphasis on quality monitoring, billing accuracy, and research, but it has often resulted in redundant documentation and inefficient workflows [2-6]. As a result, physicians have greatly increased interactions with a variety of health care information technologies (HITs), with their interactions commonly encompassing a combination of direct clinical and nonclinical goals [7]. For example, outpatient physicians now spend up to twice as much time interacting with EHRs as they do with patients [8,9].

Concurrently, symptoms of burnout, including emotional exhaustion, have risen to epidemic proportions, now affecting over 500,000 US physicians and costing the US health care system $4-5 billion annually [10-12]. Fundamentally, medicine is a human-centered endeavor, and technology can enable or distract from this focus. Unfortunately, almost 50% of physicians believe they spend an excessive amount of time on clerical tasks, and many physicians believe EHRs contribute to burnout [13-15]. High EHR task load, time spent on EHRs, and automated in-basket messages have been associated with greater emotional exhaustion [16-21]. Furthermore, the National Academy of Medicine recognizes the associations of administrative burden, technology usability, and time pressure on burnout (all of which may be attributed as EHR or HIT factors), yet the mechanisms underlying these associations are not well described [22].

One potential mechanism relating to HIT use and burnout may be frustration with technology—an emotional reaction to an obstacle preventing the fulfillment of a perceived need [23]. HIT is at risk for inducing frustration among health care workers, by virtue of its complex interfaces, frequent updates as capabilities improve, and deployment within a high-stakes environment that provides limited opportunity for dedicated training [24-26]. If frustration with technology contributes to emotional exhaustion, this would indicate an opportunity to prioritize reducing frustration through better design, training, and implementation as a mechanism to combat burnout [27].

We sought to quantify health care workers’ frustration with technology and its relationship with emotional exhaustion, after controlling for measures of work-life integration (WLI) that may indicate excessive job demands. We hypothesized that higher frustration with technology corresponds to higher emotional exhaustion.

## Methods

### Study Design and Population

This cross-sectional, observational study is a secondary analysis of the Safety, Communication, Operational Reliability, and Engagement (SCORE) survey distributed via email through the Michigan Health and Hospital Association Keystone Center in 2015 as part of their routine patient safety and quality measurement, allowing a single response per user [28,29]. All employees working 0.5 full-time equivalents or higher in any Michigan hospital for 4 consecutive weeks prior to the survey administration were invited to participate. Confidentiality was assured to the respondents, participation in the survey was voluntary, and no incentives were offered. No questions were randomized or adapted to responses in real-time, no completeness check was enforced, and reviewing of answers was allowed. No cookies, internet protocol address checks, or log files were used to exclude responses. All surveys that contained responses to the scales measuring WLI and emotional exhaustion (described below) were analyzed. Surveys with “not applicable” or missing responses to either of these two scales were excluded. This study was not considered human subjects research by Stanford University and was approved by the institutional review board at Duke University Medical Center (Pro00033155).

### Measures

The SCORE survey measures common workplace issues and work setting norms [28,29], including WLI, Emotional Exhaustion, Local Leadership, Learning Environment, Burnout Climate, Teamwork Climate, and Safety Climate scales [28,30,31]. SCORE also contains workforce engagement subscales and demographic questions (ie, number of years in specialty, job position, shift type, and length).

#### WLI and Frustration With Technology

We assessed WLI using a scale primarily focused on tangible frequencies of activities reflecting the interaction between work and personal responsibilities [28,29,32,33]. Each WLI item begins with the phrase “During the past week, how often did this occur?” The WLI items are as follows: (1) skipped a meal, (2) ate a poorly balanced meal, (3) worked through a shift with no breaks, (4) arrived home late from work, (5) had difficulty sleeping, (6) slept less than 5 hours in a night, and (7) changed personal/family plans due to work.

This WLI scale was originally validated as a 7-item scale as described above (Cronbach α=.79 in a validation study [29] and α=.81 in the current data set) and later updated with an additional eighth item assessing the frequency that one “felt frustrated with technology” as a key indicator of the ability of technology to facilitate efficient workflows and minimize conflicts between work and personal responsibilities (Cronbach α=.83 in a validation study [28] and α=.81 in the current data set). The full 8-item WLI scale is the current standard, but for the purposes of this study, we calculated WLI scores using the previously validated 7-item WLI scale, separately considering the additional item relating to frustration with technology as our primary independent variable of interest.

Each item spans a 4-point Likert scale (“rarely or none of the time,” “some or a little of the time,” “occasionally or a moderate amount of time,” and “all of the time”). For ease of interpretation, when assessing global correlations, we transposed the mean score of the 7 WLI items onto a scale of 0 to 100 and reversed the valence, with 100 indicating a favorable score (high WLI) and 0 indicating a poor score (low WLI). We similarly transposed frustration with technology onto a scale of 0 to 100, with 100 indicating a poor score (high frustration) and 0 indicating a favorable score (low frustration). For a secondary analysis comparing aggregated scores by work setting, we divided work settings into four quartiles based on the mean frustration with technology score.

#### Emotional Exhaustion

The 5-item Emotional Exhaustion scale (Cronbach α=.92) of the SCORE survey is composed of 4 items adapted from the Emotional Exhaustion subscale of the Maslach Burnout Inventory (*r*=0.96-0.98 with the 9-item scale) and a fifth item developed to align with the job demands-resources model of burnout [34-36]. This scale has been validated for use among health care workers as a burnout metric (eg, “Events in this work setting affect my life in an emotionally unhealthy way”), with demonstrated content, internal consistency, and consequence validity for this purpose [28-31,37]. Responses span a 5-point Likert scale from “disagree strongly” to “agree strongly.” We calculated each individual’s emotional exhaustion score by transposing the mean score of the five items onto a scale of 0 to 100, in line with a previous study [38].

The complete SCORE survey alongside derivation, scoring procedures, and reliability data for each of its scales is available on the internet [39].

### Classifications

Individual responses were also categorized by their specific work setting, based on self-reported work location, such as St. Elsewhere Hospital 5 South, Pleasantview Pediatrics Clinic, and Mercy Health Systems Billing Department (fictional names). Each work setting thus reflects a grouping of respondents who work together as a team, regardless of each respondent’s individual role. Work settings were classified as direct patient care (clinical) or indirect patient care (nonclinical, including administrative or billing departments). Work settings providing direct patient care were further classified as either intensive/emergency or acute; surgical or medical; and inpatient, outpatient, or mixed inpatient/outpatient. To maintain confidentiality and reduce risk of response bias from small samples, respondents from work settings with fewer than 5 total respondents were excluded from correlation and regression analyses.

### Statistical Analysis

Descriptive statistics are presented as mean and SD values, or frequencies and percentages as appropriate. We compared group means by performing two-tailed *t* tests. We evaluated agreement in frustration with technology scores within work settings by using weighted Cohen kappa agreement analysis. As a first step insight-generation analysis, we evaluated Pearson correlations among survey items by using mean scores aggregated by work setting and weighted by the number of responses, avoiding the assumptions of identically distributed observations across work settings and of nested results (eg, health care workers within work settings) [40]. In our primary analysis, we evaluated the independent relations between frustration with technology score, other measures of WLI, and the outcome of emotional exhaustion by using a single mixed-effect generalized linear regression model, with work setting as random intercept, and job position, number of years in specialty, and work setting classifiers as fixed effects. We also performed, as a sensitivity analysis, a secondary validation to control for any available potential confounding factor that the primary regression may have omitted. We leveraged the statistical machine learning method lasso [41,42] to select relevant covariates from a large set of 36 potential covariates and re-ran our regression. Analyses were performed using Stata/IC software (version 15.1; StataCorp LLC). We used simple Bonferroni correction to account for multiple comparisons. With a total of 11 comparisons (8 independent items in the regression model, plus 3 *t* tests of adjacent quartiles) and a desired family-wise error rate of <0.05, two-tailed *P* values <.0045 were considered statistically significant.

## Results

Of the 23,853 distributed surveys, 16,797 were returned (70.4% response rate). Of these, 915 indicated that technology use was “not applicable” to them and 377 had incomplete responses, resulting in 15,505 complete responses for further analysis. Descriptive statistics are presented in Table 1. The most frequently represented positions among all respondents (N=15,505) were nurses (n=4316, 27.8%), technologists and technicians (n=1890, 12.2%), and administrative support personnel (n=1800, 11.6%). The majority of respondents (n=10,284, 66.3%) reported 5 or more years in their current specialty. Nearly half (n=7286, 47.0%) of the respondents were from units not providing direct patient care, 9.0% (n=1398) were from units providing intensive or emergency care, and 10.0% (n=1559) were from units providing surgical care. Of the 1140 work settings represented, 818 (71.8 %) had 5 or more unique respondents and were included in regression analyses.

**Table 1:** Characteristics of survey respondents (N=15,505) from 1140 different work settings (818 work settings had 5 or more respondents).

Characteristic			Participant, n (%)
**Position**			
	Nurse		4316 (27.8)
	Technologist/Technician		1890 (12.2)
	Admin support		1800 (11.6)
	Admin/Manager		1238 (8.0)
	Clinical support		839 (5.4)
	Therapist		696 (4.5)
	Nurses’ aide		626 (4.0)
	Physician		431 (2.9)
	Environmental support		288 (1.9)
	Pharmacist		226 (1.5)
	Physician assistant		105 (0.7)
	Other		3050 (19.7)
**Years in specialty**			
	0-2		3056 (20.0)
	3-4		1933 (12.7)
	5-10		3374 (22.1)
	11-20		3684 (24.1)
	21 or more		3226 (21.1)
**Setting**			
	Indirect patient care		7286 (47.0)
	**Direct patient care**		8219 (53.0)
		Acute care	6821 (44.0)
		ICU^a^	1398 (9.0)
		Medical	6660 (43.0)
		Surgical	1559 (10.0)
		Inpatient	4344 (28.0)
		Mixed	3045 (19.6)
		Outpatient	830 (5.4)
**Shift**			
	Day		10,979 (70.8)
	Night		2250 (14.5)
	Swing		817 (5.3)
	Other		1214 (7.8)
**Shift length (hours)**			
	8		7889 (50.9)
	10		1272 (8.2)
	12		4091 (26.4)
	Flexible		874 (5.6)
	Other		1211 (7.8)
**Frustration with technology**			
	Rarely		6310 (40.7)
	A little		4130 (26.6)
	Occasionally		2815 (18.2)
	Always		2250 (14.5)

^a^ICU: intensive care unit.

**Figure 1:**
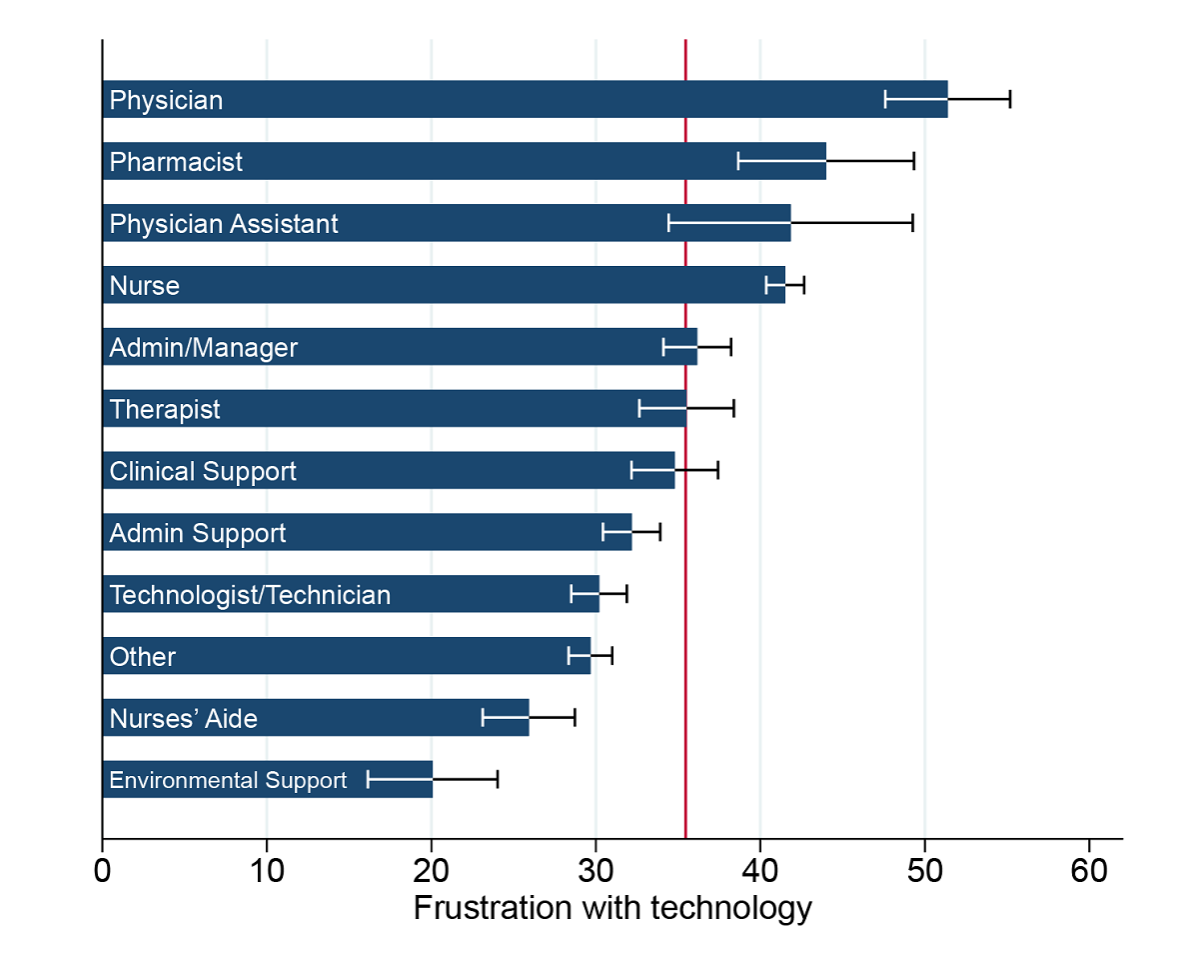
Frustration with technology scores by job position. Data shown as mean values and 95% confidence limits of the mean, with the reference line at a population mean of 35.03.

WLI scores ranged from 0 to 100 (higher score favorable), with a mean score of 68.4 (SD 23.4) and median score of 71.4 (IQR 52.4-85.7). Separately, frustration with technology was reported as “none/rarely” by 6310 (40.7%) of the 15,505 respondents, “some/a little” by 4130 (26.6%), “occasionally/moderate” by 2815 (18.2%), and “all the time” by 2250 (14.5%) respondents. Frustration with technology scores ranged from 0 to 100 (lower score favorable), with a mean score of 35.0 (SD 35.9), and the score was higher among direct clinical care providers (mean 36.8, SD 36.4) than indirect providers (mean 32.9, SD 35.3; *P*<.001). The distribution of frustration with technology scores among 818 work settings, with corresponding WLI scores, is shown in Multimedia Appendix 1. The mean frustration with technology scores by job type are presented in Figure 1; the highest scores were reported by physicians, pharmacists, physician assistants, and nurses. Agreement in frustration with technology within work settings was low, with a combined weighted Cohen κ=0.04. Emotional exhaustion scores ranged from 0 to 100 (lower score favorable), with a mean score of 41.6 (SD 30.7).

Work setting correlations among frustration with technology, WLI items, and emotional exhaustion responses are illustrated in Multimedia Appendix 2 and tabulated in Multimedia Appendix 3. Frustration with technology was positively correlated with the full Emotional Exhaustion scale (*r*=0.35) as well as each individual item on the scale (*r*=0.29-0.36). The reverse-transposed WLI scale was negatively correlated with the Emotional Exhaustion scale and its individual items (*r*=–0.55 to –0.63). Each individual WLI item was correlated with the Emotional Exhaustion scale and its individual items, with the smallest correlations for “arrived home late from work” (*r*=0.32-0.41) and the largest correlations for “had difficulty sleeping” (*r*=0.57-0.65).

In our primary analysis, frustration with technology and six of the seven WLI items were independently related to emotional exhaustion in multivariable modeling, each associating with a 0.34- to 2.06-point increase in the emotional exhaustion score for each 10-point change (on a 100-point scale), as shown in Table 2. Frustration with technology was associated with a 1.23-point (95% CI 1.07-1.38) increase in the emotional exhaustion score with each 10-point change, and it was second only to *difficulty sleeping* out of the WLI items most strongly associated with emotional exhaustion. For example, an increase in frustration with technology score from 30 to 40 would correspond to a 1.23-point increase in the emotional exhaustion score, all else being equal. The frequency of sleeping less than 5 hours a night was the only WLI item that was not independently associated with emotional exhaustion. Results were similar when stratified by direct patient care versus indirect patient care. The results of our sensitivity analysis, as shown in Multimedia Appendix 4, are aligned with our primary regression model.

**Table 2:** Frustration with technology and work-life integration as independent predictors of emotional exhaustion.

Work-Life Integration scale item		β^a^	95% CI	*P* value
Felt frustrated by technology		1.23	1.07 to 1.38	<.001
**During the past week, how often did this occur?**				
	Had difficulty sleeping	2.06	1.88 to 2.25	<.001
	Changed personal/family plans because of work	.99	0.80 to 1.18	<.001
	Worked through a day/shift without any breaks	.87	0.69 to 1.05	<.001
	Arrived home late from work	.82	0.64 to 1.00	<.001
	Ate a poorly balanced meal	.67	0.49 to 0.85	<.001
	Skipped a meal	.34	0.14 to 0.54	.001
	Slept less than 5 hours in a night	.01	–0.18 to 0.19	.94

^a^Estimates via a single multivariable mixed model with work setting as random intercept. Beta coefficients reflect the change in emotional exhaustion score for each 10-point increase in frustration or work-life integration item (100-point scale) evaluated among 12,528 respondents in 818 work settings, adjusted for job type, years of experience, patient care type (intensive care vs not, surgical vs not, inpatient vs not), and direct patient care vs indirect patient care.

**Figure 2:**
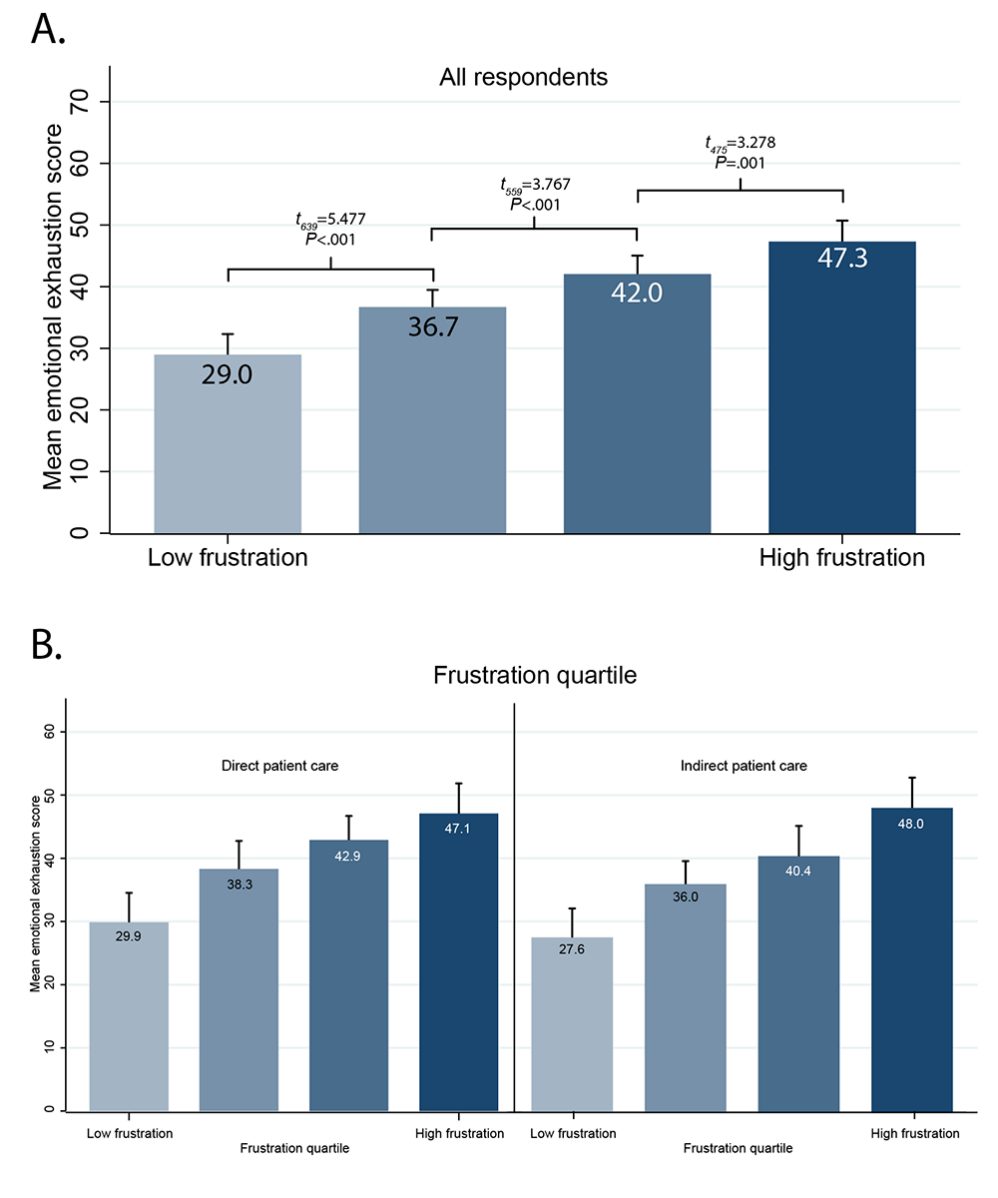
Emotional exhaustion scores, stratified by quartile of the technology frustration scores for each work setting, shown for all respondents (A) and stratified by direct patient care versus indirect patient care (B). Data are shown as mean values and upper 95% confidence limits, with results of *t* tests of adjacent quartiles.

Our secondary analysis relating frustration with technology and emotional exhaustion aggregated within work settings is shown in Figure 2. Work settings with higher mean frustration with technology scores (distributed into quartiles) had higher emotional exhaustion scores. Mean emotional exhaustion scores ranged from 29.0 (SD 18.7, 95% CI 25.6-32.3) in the lowest quartile to 47.3 (SD 19.1, 95% CI 43.9-50.7) in the highest quartile. Results were similar when stratified by direct patient care versus indirect patient care.

## Discussion

This study found that frustration with technology varies with health care worker role and among individuals within work settings. Frustration with technology and 6 of 7 WLI items are independently associated with emotional exhaustion. Although frustration with technology was higher among direct clinical providers, similar relationships with emotional exhaustion were apparent for respondents engaged in direct patient care compared to those engaged in indirect patient care.

These results highlight and build on the evidence relating health care workers’ user experience with well-being outcomes [16,43,44]. HIT differs from consumer technology in that the purchaser (eg, health care administration) often has different priorities than the end users (health care workers), making user experience less incentivized as a driver in technology development. Although the purchaser’s incentives (eg, improving patient care quality and communication, facilitating accurate reimbursement, and developing research and analytics infrastructure) are undeniably important, our findings highlight the need for attention to user experience with HIT [45]. Recently, physician-reported EHR usability was reported to be in the bottom 10th percentile relative to system usability scores in other industries, but physicians reporting better EHR usability had lower odds of burnout [26].

Frustration with technology may arise from discrepancies between expectations and reality [24]. Particularly in settings in which EHRs or other HITs were rapidly implemented, health care workers’ expectations of the benefits of these technologies may not have been accurately set, features may not have been fully explained, or efficient use of the interface may not have been taught [46,47]. The Health Information Technology for Economic and Clinical Health (HITECH) Act of 2009 that incentivized the adoption of HIT was effective at encouraging transition to EHRs, but rapid adoption may have precluded adequate attention to end-user input, development of interoperability standards, setting realistic expectations, or education on the use of new technologies [1,48-50].

Emotional exhaustion, the key construct of burnout evaluated in this study, reflects a depleted state arising from excessive demands, continuous stress, or insufficient resources [34,35,51-54]. If an EHR requires many clicks or otherwise inefficient workflows to accomplish tasks, this would translate to increased job demands. Alternatively, if inadequate training results in a health care worker not knowing how to use the interface effectively, this lack of knowledge translates to insufficient resources to use the available tools. In both cases, frustration with technology may develop as an indicator of imbalanced job demands and resources.

In our primary analysis, frustration with technology was found to be associated with burnout independently of other markers of WLI. This association was not as strong as the one observed for difficulty sleeping, consistent with observations that sleep disturbance may itself be an indicator of psychological distress [55]. However, it was similar in magnitude to the associations for other items reflecting excessive workload or workplace inefficiencies, such as changing personal/family plans because of work, working a shift without any breaks, or arriving home late from work. This pattern of findings suggests that frustration with technology is more closely related to an imbalance between job demands and resources, and it is less similar to psychological distress markers.

At the work-setting level, average frustration with technology was significantly associated with higher emotional exhaustion, which indicates a potential climate-like effect of frustration with technology, similar to that observed for WLI [28]. However, agreement among respondents from the same work setting was low, suggesting that much of frustration with technology is rooted at the individual level rather than the work-setting level (ie, the individual’s experience with technology rather than problems with the technology itself). Even work settings with the highest average frustration with technology scores still contained individuals reporting low frustration, suggesting against the notion that frustration is inevitable or inextricably linked to HIT. Different individuals may have vastly different experiences using the same technology, related to their specific worker tasks, training, personal expectations, comfort with the technology, and acceptance of change [56].

Of note, frustration with technology was only moderately correlated with frustration with one’s job in general, indicating that frustration with technology can occur without generalized job frustration and that individuals may remain motivated to take action to reduce their frustrations. This pattern of findings suggests that ensuring all individuals receive adequate support and training may be effective at reducing frustration with technology, rather than focusing only on making extensive changes to the technology or the user interface itself.

Several practical steps may be taken to reduce the frequency of frustration with technology among health care workers. Although details will require tailoring to specific settings, tasks, and technologies available, our findings suggest an approach of educating and supporting individuals experiencing the most frequent frustrations. Recent studies have described the use of supplemental EHR training to improve comfort with technology and ability to work more efficiently [57,58]. Interventions at the work-setting level may also include measuring and reducing individual workloads, such as employing scribes to assist with clinical documentation, transitioning to team-based documentation and inbox management, or automating data-entry tasks [59-72]. Finally, interventions to reduce workloads placed on the system could have the broadest benefits, but it would likely require changes to current payment structures that promote lengthy documentation and labor-intensive payment authorizations [73].

This study must be interpreted in the context of its design. As a cross-sectional, observational study, it cannot determine causality or directionality of the observed correlations (ie, whether frustration with technology induces burnout, burnout amplifies frustrations, and/or an external factor influences both). Although we were able to adjust for many potential confounders in our regression model, it is possible that residual confounding from unobserved variables such as age or prior experience with technology remains. Emotional exhaustion was also evaluated, but it does not capture other manifestations of burnout such as depersonalization. The 100-point emotional exhaustion scale we used differs from scores generated by other burnout instruments and cannot be used to directly compare effect sizes from studies using different instruments; however, methods to approximate estimates from disparate instruments have been previously described [74]. Although we used the 7-item WLI scale in this study, the 8-item version is commonly used, internally consistent (Cronbach α=.81 for this sample), and appears to add a unique element to the WLI assessment as evidenced by the results presented here.

The response rate of over 70% compares favorably with other studies of this magnitude and exceeds commonly accepted thresholds for survey-based research; however, there may remain some sampling bias, and it is possible that physicians in particular were relatively underrepresented in this sample [75,76]. Similarly, although the survey was confidential, as with any self-reported measures, the responses may be susceptible to recall bias or social desirability bias. The survey respondents reflect a wide variety of health care roles and work settings, with varying interactions with technology. Furthermore, even though many of the direct and indirect patient care roles included in this study heavily feature EHR use, these and other respondents may have referenced other use of technology in their responses, and we did not conduct interviews to further characterize their responses. Prior qualitative research has found that specific drivers of HIT frustration vary among individuals, tasks, and settings [47,77]. Our findings are thus reflective of the overall conceptual relationship between frustration and emotional exhaustion but are unable to provide conclusions regarding any particular piece of technology or source of frustration. Additional research will be necessary to further delineate the specific sources and scope of frustration with technology across health care worker roles, as these data may provide more granular insights of potential interventions to reduce frustrations. Although this survey was administered within a single US state, the Michigan Health and Hospital Association Keystone Center includes all 175 hospitals from 20 health systems within Michigan, making our results likely to be generalizable to community and academic hospitals across the United States.

It remains unknown whether reducing frustrations with technology through improved training, updated interfaces, or redistributed tasks will be effective in reducing burnout. Longitudinal observational studies may enhance our understanding of the directionality of these relationships, but prospective trials will be needed to fully evaluate the effect of interventions to improve health care worker user experience and well-being.

In conclusion, frustration with technology and difficulty sleeping were the biggest WLI factors associated with emotional exhaustion across direct and indirect patient care settings. Interventions designed to reduce health care workers’ frustration with technology and improve other aspects of WLI may be effective strategies to reduce burnout among health care workers.

## Appendix

Multimedia Appendix 1 Frustration with technology and work-life integration scores for 818 individual work settings, ordered by mean frustration with technology scores. Lower panel shows mean work-life integration scores for the corresponding units, suggesting a negative unadjusted correlation. Data are shown as mean values and 95% confidence limits.

Multimedia Appendix 2 Work-setting frustration with technology scores in relation to emotional exhaustion scores. For ease of interpretation, values are shown as means and 50% confidence limits for each work setting (total of 818 work settings with 5 or more respondents). CLM: confidence limits of the means.

Multimedia Appendix 3 Item-level correlations with emotional exhaustion items for 818 work settings.

Multimedia Appendix 4 Sensitivity analysis. Multivariable regression with independent variables chosen using lasso.

## Abbreviations

EHR: electronic health record
HIT: health information technology
HITECH: Health Information Technology for Economic and Clinical Health
SCORE: Safety, Communication, Operational Reliability, and Engagement
WLI: work-life integration
