# Supplementary figures and images for: Frustration With Technology and its Relation to Emotional Exhaustion Among Health Care Workers: Cross-sectional Observational Study
Source: J Med Internet Res. 2021 Jul 6;23(7):e26817. doi: 10.2196/26817 (PMC8292941; doi:10.2196/26817)

MULTIMEDIA APPENDIX 1


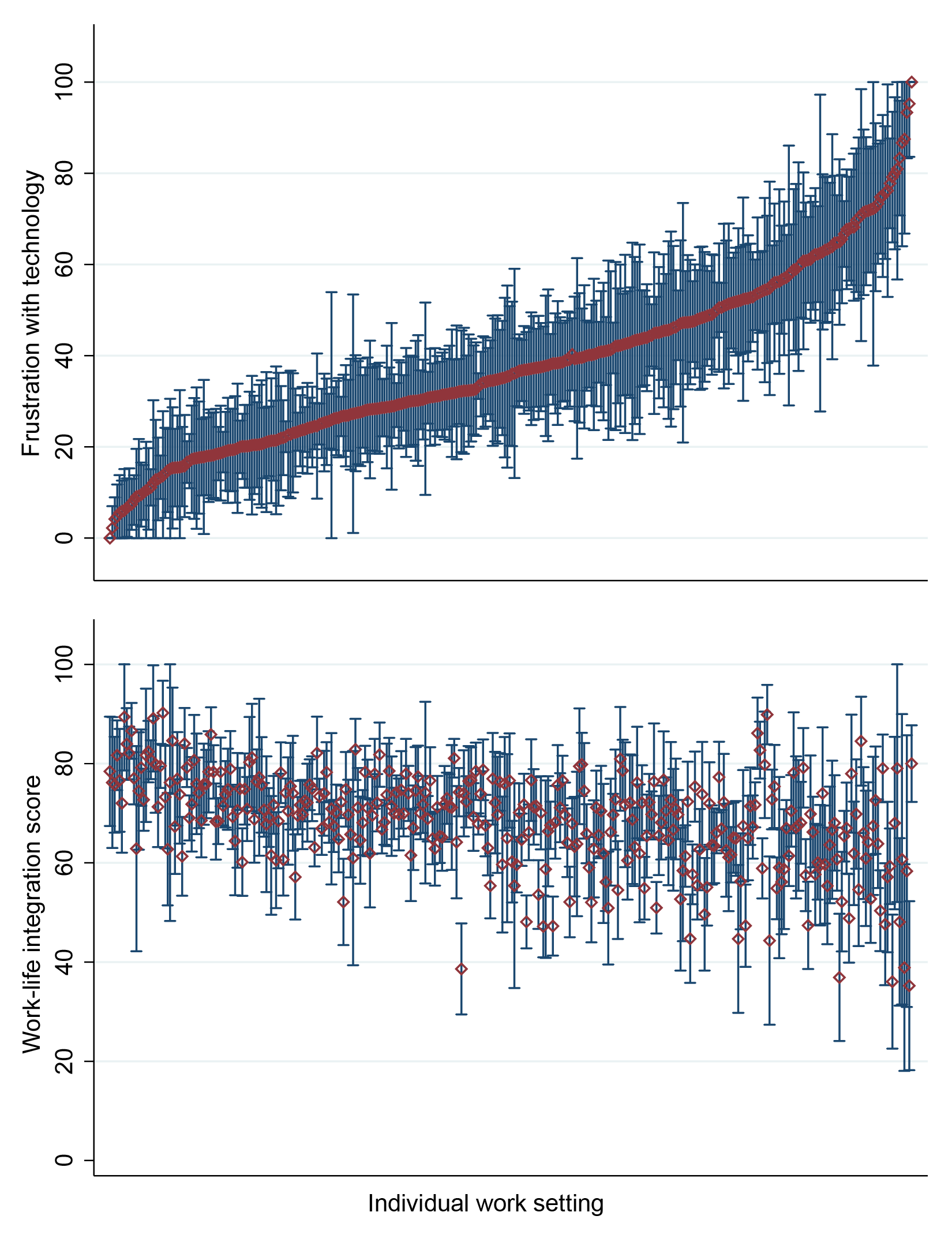

Supplement: Multimedia Appendix 1 [file jmir_v23i7e26817_app1.docx]

MULTIMEDIA APPENDIX 2


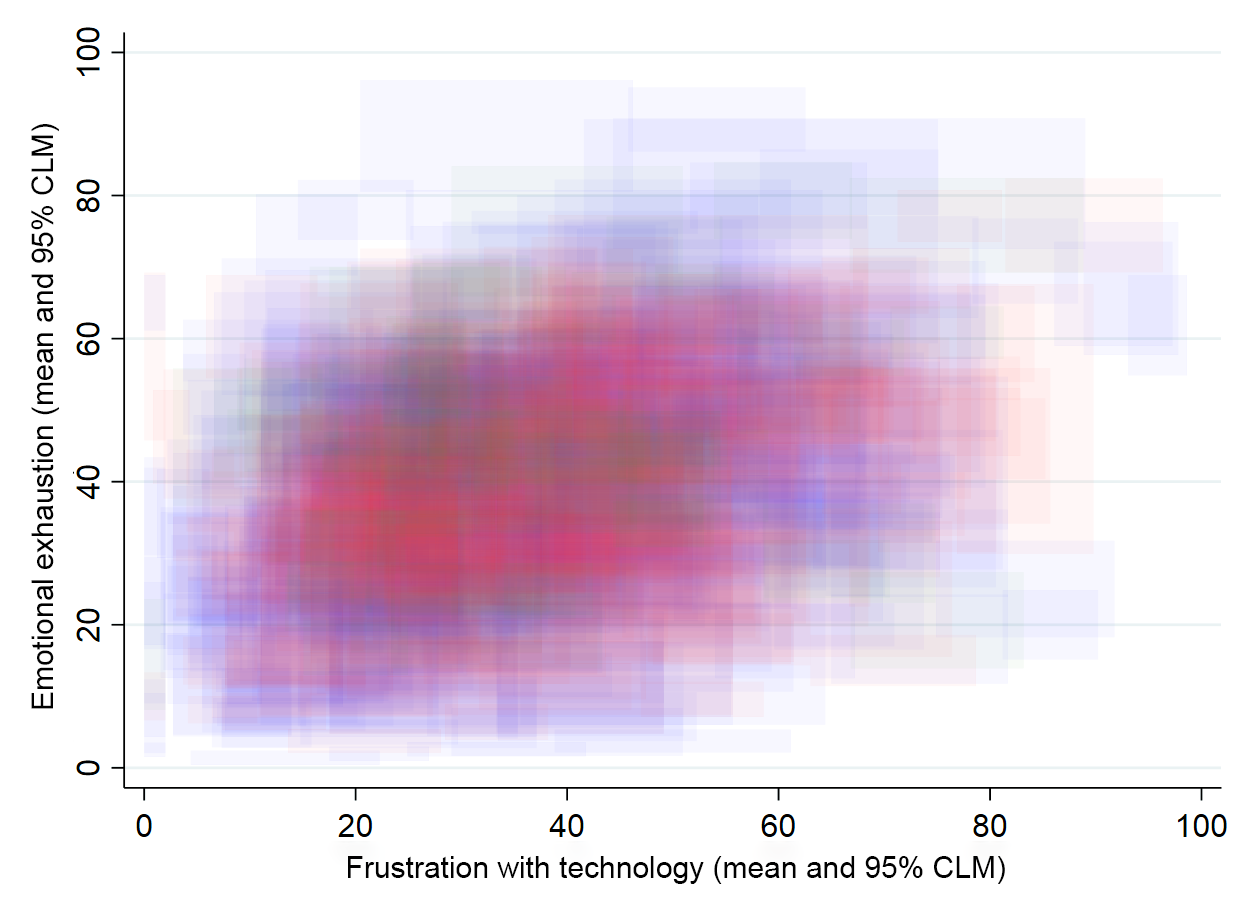

Supplement: Multimedia Appendix 2 [file jmir_v23i7e26817_app2.docx]
