# Supplementary material for: Frustration With Technology and its Relation to Emotional Exhaustion Among Health Care Workers: Cross-sectional Observational Study
Source: J Med Internet Res. 2021 Jul 6;23(7):e26817. doi: 10.2196/26817 (PMC8292941; doi:10.2196/26817)
Supplement: Multimedia Appendix 3 [file jmir_v23i7e26817_app3.docx]

MULTIMEDIA APPENDIX 3

|  | *Work setting mean (SD)* | Emotional exhaustion scale | Events in this work setting affect my life in an emotionally unhealthy way. | I feel burned out from my work. | I feel fatigued when I get up in the morning and have to face another day on the job. | I feel frustrated by my job. | I feel I am working too hard on my job. |
| --- | --- | --- | --- | --- | --- | --- | --- |
| *Work setting mean (SD)* |  | ***39.59 (15.78)*** | *38.67 (17.05)* | *38.07 (17.28)* | *38.63 (17.07)* | *42.36 (17.09)* | *40.39 (15.91)* |
|  |  |  |  |  |  |  |  |
| **Frustration with technology** | ***35.26 (17.53)*** | 0.35 | 0.33 | 0.29 | 0.30 | 0.36 | 0.34 |
|  |  |  |  |  |  |  |  |
|  |  |  |  |  |  |  |  |
| **Work-life integration scale** ^a^ | ***70.18 (11.03)*** | -0.63 | -0.60 | -0.60 | -0.63 | -0.55 | -0.60 |
| Skipped a meal | *25.20 (15.14)* | 0.48 | 0.46 | 0.44 | 0.47 | 0.42 | 0.47 |
| Ate a poorly balanced meal | *32.76 (14.21)* | 0.51 | 0.48 | 0.49 | 0.53 | 0.45 | 0.47 |
| Worked a shift without breaks | *29.62 (17.45)* | 0.42 | 0.40 | 0.39 | 0.39 | 0.38 | 0.42 |
| Arrived home late from work | *33.43 (16.57)* | 0.39 | 0.36 | 0.38 | 0.37 | 0.32 | 0.41 |
| Had difficulty sleeping | *34.94 (13.40)* | 0.64 | 0.59 | 0.61 | 0.65 | 0.57 | 0.59 |
| Slept less than 5 hours in a night | *28.34 (13.10)* | 0.53 | 0.49 | 0.51 | 0.55 | 0.45 | 0.48 |
| Changed personal/family plans | *24.46 (13.05)* | 0.56 | 0.53 | 0.54 | 0.56 | 0.50 | 0.52 |
|  |  |  |  |  |  |  |  |
| ^a^ Work-life integration scale is reverse-scaled such that higher scores are more favorable.  All *P* values < .001 | | | | | | | |
