# Supplementary material for: Frustration With Technology and its Relation to Emotional Exhaustion Among Health Care Workers: Cross-sectional Observational Study
Source: J Med Internet Res. 2021 Jul 6;23(7):e26817. doi: 10.2196/26817 (PMC8292941; doi:10.2196/26817)
Supplement: Multimedia Appendix 4 [file jmir_v23i7e26817_app4.docx]

MULTIMEDIA APPENDIX 4

|  | β | 95% CI | *P* value |
| --- | --- | --- | --- |
| Felt frustrated by technology | 1.25 | 1.11-1.39 | <.001 |
| During the past week, how often did this occur? |  |  |  |
| Had difficulty sleeping | 2.09 | 1.92-2.26 | <.001 |
| Changed personal/family plans because of work | 1.08 | 0.90-1.26 | <.001 |
| Worked through a day/shift without any breaks | 0.81 | 0.65-0.98 | <.001 |
| Arrived home late from work | 0.63 | 0.46-0.80 | <.001 |
| Ate a poorly balanced meal | 0.74 | 0.57-0.92 | <.001 |
| Skipped a meal | 0.29 | 0.09-0.49 | .004 |
| Slept less than 5 hours in a night | 0.12 | -0.06-0.30 | .19 |
| Estimates via a single multivariable mixed model with work setting as fixed effect. Beta coefficients reflect the change in emotional exhaustion score for each 10-point increase in frustration or work-life integration item (100-point scale).  N=12,528 respondents in 818 work settings. Also adjusted for job type, shift (day vs. night), typical shift length, years of experience, WalkRounds frequency, WalkRounds with feedback frequency, WalkRounds participation, patient care type (ICU vs. not, surgical vs. not, inpatient vs. not), and direct patient care vs. indirect patient care. | | | |
